# Micro- and Nanoplastics as Emerging Threats to Both Terrestrial and Aquatic Animals: A Comprehensive Review

**DOI:** 10.3390/vetsci12080688

**Published:** 2025-07-23

**Authors:** Munwar Ali, Chang Xu, Kun Li

**Affiliations:** College of Veterinary Medicine, Nanjing Agricultural University, Nanjing 210095, China; drmunwarali06@gmail.com (M.A.);

**Keywords:** plastics, animals, plants, environment, hazards

## Abstract

Micro- and Nanoplastic (MNP) pollution is an emerging challenge globally, posing a significant threat to both aquatic and terrestrial ecosystems worldwide. This review critically examines the sources, exposure routes, and impact of plastics, with particular focus on implications for the livestock sector. MNPs enter animals’ bodies primarily through ingestion of contaminated feed and water, inhalation, and dermal exposure, subsequently accumulating in various organs, disrupting physiological functions. Notably, MNPs facilitate the horizontal transfer of antimicrobial resistance genes (ARGs), exacerbating the global challenge of antimicrobial resistance (AMR). In agricultural environments, sources such as organic fertilizers, wastewater irrigation systems, surface runoff, and littering contribute to soil contamination, adversely affecting plant growth and soil health, which in turn compromises feed quality and ultimately animals’ productivity. This review synthesizes current evidence demonstrating how MNP exposure impairs animal production, reproduction, and survival, and highlights the interconnected risks to food safety and ecosystem health. The findings call for the urgent need for comprehensive research under controlled conditions to underscore the fine details regarding mechanisms of MNP toxicity and to inform effective mitigation strategies. Addressing MNP pollution is crucial for safeguarding animal health, ensuring sustainable livestock production, and promoting environmental sustainability and integrity.

## Simple Summary

Micro- and Nanoplastics (MNPs), tiny particles found everywhere in the environment, pose an ever-growing threat as they reach the GITs of terrestrial and aquatic animals. These particles contaminate feed and water, enter the body, and spread to different organs, harming wildlife, livestock, and crops. This review aims to show how MNPs damage different organs of animals, reduce fertility, and also lead to feed shortages by stunting plant growth and reducing soil fertility. These small particles also carry notorious chemicals and lead to increased antimicrobial resistance by facilitating horizontal gene transfer (HGT) between bacteria, hence posing an increased risk of diseases to the livestock sector. This study underscores how these threats are linked to animals and environmental well-being. By demonstrating these risks, this review urges measures to curb plastic pollution and encourages further research to develop preventive and control strategies.

## 1. Introduction

Improper disposal, fragmentation, and/or degradation of plastics have increased the number MNPs in the environment, including both primary plastics intentionally produced at the micro- and nanoscale level and secondary plastics formed through the degradation of larger plastic items [1,2,3]. The emergence of these minute plastic particles has raised concerns due to their capacity to accumulate in water bodies and their potential adverse effects on aquatic organisms [4]. Therefore, their pollution has become an increasingly concerning global issue, along with its detrimental impacts on ecosystems [5]. While the effects of MNPs are well-documented, the presence and potential risks of MNPs in the environment have recently gained significant attention [6]. These small plastic particles, measuring less than 5 mm (MPs) and 100 nm (Nanoplastics) [7,8], have become pervasive contaminants in aquatic ecosystems, posing potential risks to aquatic organisms and overall ecosystem health.

Primary MPs are mass-produced for manufacturing and domestic applications, which include exfoliating facial scrubs, toothpaste, detergents, other personal care products, abrasive cleaning agents, plastic powder for molding, and synthetic clothing (nylon/polyester) [9,10], while paints, adhesives, electronics, etc., are responsible for NP release [11]. Secondary MNPs are formed by fragmentation of macroplastics (200–1000 µM) through shear forces [9,10], and account for 70–80% of all plastic released into the environment, while primary MPs contribute only 15–30% [12,13]. Additionally, synthetic microfibers are often the most commonly reported form of microplastics in environments, from soil to aquatic systems (e.g., oceans, rivers, shorelines, and lakes) [14,15]. Approximately 63% of textile fibers produced are synthetic (e.g., polyester, nylon), and over 42 million tons of synthetic fibers are produced each year by the clothing industry [16], with polyester dominating production (approximately 80%) [17]. As such, Boucher (2017) [18] estimated that of all primary microplastics in the world’s oceans, 35% arise from the laundering of synthetic textiles, an estimated 2–13 million tons per year globally. Without an instant mitigation strategy, plastic pollution in the oceans will be more than 6 million tons by 2040 [19]. Increased plastic accumulation in the environment is due to low degradation rates in addition to unsustainable use and improper disposal [20]. Although the impacts of these ever-present plastics are not fully understood, interest is shifting toward the plastics’ associated hazards in animals (especially food animals) and the environment.

MNPs enter the water bodies through domestic waste, sewage treatment plants (STPs), industrial effluents, stormwater, estuaries, riverine transport, surface runoff, wind currents, and disposal practices [21,22]. MNPs are present as fragments, pellets, fibers, films, granules, and Styrofoam that vary with surface–mass area ratios [23]. The chemical variations found in STPs include polyethylene (PE), polystyrene (PS), and polypropylene (PP), which tend to float, while polyethylene terephthalate (PET) and polyvinyl chloride (PVC) are more likely to sink due to their density [24,25]. The presence of MNPs in the air, water, and food can cause ecotoxicological issues [26]. Research results indicated that MNPs fostered alterations in gene expressions, reduced fertilization efficiency [27], increased oxidative damage [28], and liver abnormalities in aquatic animals like zebrafish, rainbow trout, mollusks, mussels, oysters, etc. The hazardous effects of plastic particles have been proven in mice, sea birds, chickens, dogs, cats, and pigs [29,30]. In plants like wheat, corn, oats, tobacco, millet, etc., MNPs cause reduced biomass [31], decreased germination rate, and reduced growth [32]. MNPs also act as vectors to adsorb tenacious organic pollutants, trace metals, and harmful additives at rates that are multiple times higher than natural sediments [33]. The presence of MNPs in STPs impacts microbial communities, inhibits sludge hydrolysis, and accumulates acids [34].

MNPs indicate a higher risk because, due to their small size, these plastics are quickly able to penetrate cells and tissues. The complexity of MNP separation and identification, as well as their abundance in the environment, has been largely disregarded until now. As a result, the physical presence and health risks posed by MNPs may be underestimated. Although many studies have demonstrated the accumulation of MNPs in aquatic organisms, evidence for bioaccumulation and health implications in terrestrial livestock remains limited and mostly relies on extrapolation from laboratory models. Furthermore, most existing studies have rarely examined the complex interaction between MNPs and other environmental contaminants, nor do they address the long-term, low-dose exposures typical in agricultural settings.

This review aims to provide current perspectives on MNPs concerning (1) the occurrence of issues posed by MNP pollution, including their transmutation routes into animals via ingestion, inhalation, and hide/skin exposure, as well as indirect entry through feed; (2) the toxicokinetic and toxicodynamic of MNPs, highlighting their role as transporters for antimicrobial resistance genes (ARGs), hazardous chemicals, and other pollutants; (3) the impact of MNPs on plant health, aquatic organisms, and livestock productivity, interconnecting human sources of plastic pollution, animals, and environmental health; (4) managemental strategies to mitigate MNPs through current understandings. By integrating recent advances on MNPS-mediated antimicrobial resistance and their effects on livestock health and productivity, this review provides novel perspectives that bridge environmental science and veterinary medicine.

## 2. Methodology

A rigorous search strategy was employed to retrieve articles from diverse databases. The literature search was conducted using the keywords “Micro-plastics” and “Nanoplastics”, “animals”, “human-based sources of plastics”, and “plastics in the environment” across multiple search engines, including Google Scholar, NCBI, Scopus, ScienceDirect, and PubMed. Sticking to the title, initially, 250 research and review articles were studied, and finally, 176 articles were included in this review. Use of the latest literature throughout the process remained our topmost priority. The synthesis of findings involved a thematic analysis, categorizing and summarizing key themes and trends identified across the selected studies, providing a coherent narrative of the current state of knowledge in the field after intense selection to divert the attention of readers toward this multidisciplinary issue.

## 3. Non-Polymeric Components in Plastics: Key Additives and Their Functions

Plastics are widely valued for their versatile mechanical and physicochemical properties, but many plastic products only achieve their specific functionalities through various additives. Flame retardants, stabilizers, and plasticizers are among the most commonly used, each serving a distinct purpose in enhancing the performance of plastics [35]. For instance, brominated flame retardants (BFRs), which are halogenated and can be either hydrophobic or reactive, are used to improve the fire resistance of plastic products. More than 75 different BFRs are currently in use, although some have been restricted due to their environmental and health impacts. As a result, newer types, such as brominated polymers and reactive BFRs, are gradually replacing older compounds like hexabromocyclododecane (HBCD) [36]. Similarly, plasticizers play an essential role in improving the flexibility of plastics by increasing the distance between polymer chains. This loosens the dipolar forces, allowing the chains to slide against each other more easily. Phthalates are the most widely used plasticizers, making up about 90% of PVC plastic products [37]. Another critical category of additives is stabilizers, such as antioxidants, which prevent degradation by protecting polymer chains or eliminating hydroperoxides. Hydroperoxides can otherwise degrade into radicals that damage other polymer chains, compromising the integrity of the plastic [38,39]. While these additives are crucial for enhancing the properties of plastics, some of the substances used historically have been reevaluated due to their long-term impacts. Many of these are now classified as substances of very high concern (SVHCs) or persistent organic pollutants (POPs), earning them the designation of “legacy additives” due to their persistence in the environment and associated risks [35].

## 4. Accumulation of MPs in Terrestrial and Aquatic Environments and Associated Hazards in Animals

**Figure 1:**
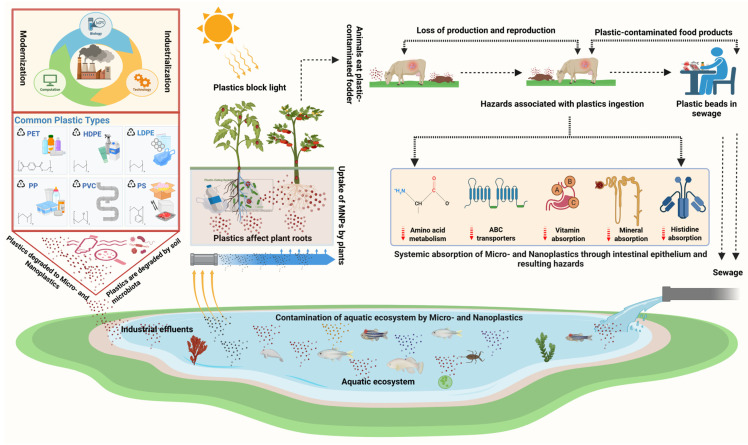
The flow of MNPs through the terrestrial and aquatic environment. MNPs enter animal bodies by grazing, consuming feed containing plastics originating from human sources, e.g., plastic utensils, drinking water, personal care products, aquatic food items, and directly from plastic appliances. The entry of plastics into animal bodies leads to production- and reproduction-associated losses.

Concerns about the incorporation of MPs into feed have grown as the number of MPs in water bodies has increased. The effect of polymers or plastics starts from the lowest level of the food web itself in the aquatic environment. MPs are the same size as plankton and grains of other organic food materials, allowing them to be consumed by a variety of organisms with various feeding strategies [40]. Additionally, the differences in density and shape of these polymers affect their behavior [41] and distribution among different compartments (surface, water column, and sediment) of the aquatic environment, influencing their availability to organisms at various trophic levels [41]. The ingestion of MPs causes several physical and biological impacts on organisms. It disrupts feeding in algae and filter-feeding organisms [42], thereby reducing the weight of the organisms and thus leading to mortality and a decrease in fertility [43]. As discussed in the earlier section, apart from the physical impacts of ingested MPs alone on organisms, adverse health effects also occur from additives, absorbed contaminants, and so on, which are carcinogenic and even capable of endocrine disruption in organisms [44]. The ingestion could be due to a failure to distinguish MPs from prey, or it could be due to the intake of lifeforms from lower trophic levels that contain these particles [45]. MPs may also adhere directly to organisms [46]. In terms of food safety, MNPs are also an emerging threat, as these particles can eventually end up in the human food chain through fish and other kinds of aquatic and terrestrial food animals (Figure 1) [47].

In livestock, mice, and poultry, MNPs can lead to enteritis, gut barrier dysfunction, gut microbiota dysbiosis, and reduced growth [48]. Similarly, hepatic inflammation, an altered cytokine profile (increased IL-6, TNF-α, and decreased IL-10), immune cell dysregulation, and metabolic disorders have been detected after the accumulation of MNPs, causing oxidative stress [48]. Reproductive toxicities and endocrine disruptions have been observed in rodents, aquatic species, and livestock, leading to reduced fertility, ovarian or testicular damage, and granulosa cell apoptosis [48,49]. Physiological damage and restricted growth have been detected in earthworms and chickens, with reduced gizzard volume and decreased foraging time due to physical obstruction and irritation by plastic particles [48]. Microbial contamination and the spread of antibiotic resistance have been reported in livestock and aquatic species, as MNPs act as vector for pathogens like bacteria, viruses, and fungi, which form biofilms on MNPs, facilitating microbial contamination and gene transfer [48,50]. Kidney, spleen, and tissue damage have been observed in many rodents and livestock species, leading to inflammation, fibrosis, loss of organ structure, and reduced tubular glands [49]. Neurological and behavioral effects have been observed in rodents and aquatic species, resulting in neuroinflammation, altered neurotransmitter levels, impaired behavioral cognition due to translocation of NPs across the blood–brain barrier, and oxidative stress [51]. Additionally, genotoxicity and epigenetic changes have been observed in rodents and aquatic species, characterized by DNA damage, altered gene expression, DNA hypomethylation, and oxidative damage [48,49]. Likewise, in different livestock, poultry, and aquatic species, accumulation of MNPS in muscle, liver, milk, eggs, and seafoods, can be a potential danger to humans through the food chain due to persistence of plastics and additives in tissues [52]. Metabolic disorders in rodents and livestock have been detected due to altered glucose and lipid metabolism, leading to increased blood glucose and cholesterol and resulting in the disruption of metabolic pathways via inflammation and oxidative stress [48].

## 5. Exposure Pathways and Associated Hazards of MNPs

### 5.1. Via Ingestion

Plastics are commonly found in atmospheric, terrestrial, and aquatic environments, and enter living organisms mostly via ingestion [53]. According to the study by Galloway (2015), ingestion is the major route for MPs entering into living organisms [54]. Particles may enter the digestive tract through contaminated feed and are absorbed by the gut lining, depending on their size and hydrophobicity. Smaller MNPs, particularly those in the nanoscale range, can pass through tight junctions among enteric epithelial cells [55]. Larger MNPs, however, are absorbed through endocytosis, a process in which cells swallow the particle. After ingestion, MNPs result in enteritis, increased gut permeability, increased oxidative damage, gut microbial dysbiosis, metabolic dysfunctions, genotoxicity, and immunotoxicity [56,57,58,59]. It has been reported that MPs, if ingested, may accumulate and cause localized toxicity by triggering and/or increasing immunological responses, thereby weakening the body’s defenses against infections and changing how energy reserves are used [60,61]. In aquatic organisms, particles are accumulated in the gill, liver, intestine, mantle, and stomach of bivalves through ingestion. The aggregation of MPs stimulates oxidative stress and imbalance of the antioxidation system [62]. The literature indicates that exposure to MPs could significantly induce inflammatory reactions by activating the NF-κB pathway in zebrafish larvae (Table 1) [63].

### 5.2. Via Inhalation

In living organisms, inhalation of MNP-contaminated aerosols leads to their entry into the respiratory tract [69]. Animals inhale MPs from a variety of sources, including environmental dust (about 272 MPs because of polluted air), industrial emissions, and synthetic garment fibers [61]. The size and shape of airborne MPs determine where they are deposited in the respiratory system. For instance, larger particles are more likely to accumulate in the top airways, where mucociliary clearance mechanisms transport them to the GIT through swallowing [70]. On the other hand, smaller MNPs can penetrate alveolar surfaces, ultimately enter the circulation, and be transported to the whole body. Outdoor MNPs can originate from the disintegration of bigger plastic trash owing to UV radiation, weathering, and physical processes, and become airborne due to wind propagation [71]. Thus, previous investigations indicated that MNP inhalation can lead to severe respiratory disorders (Table 1).

### 5.3. Through Dermal Exposure

However, although inhalation is a well-documented exposure route, research studies have proved the possibility of dermal exposure through direct contact with sources such as agricultural waste, textiles, or contaminated water and personal care products [70,74]. Injured skin or extended exposure to specific environmental conditions increases the chances of MNPs permeating skin barriers [75]. Dermal absorption depends upon particle size, surface qualities, and shape, as well as the cutaneous barrier’s health and integrity. In general, larger MNPs (>100 nm) are less likely to penetrate the stratum corneum. Smaller MNPs, particularly of size (<100 nm), can penetrate hair follicles and pass through damaged skin to reach deeper layers [76]. According to a study by Raszewska-Famielec (2022), smaller MPs or NPs may concentrate within hair follicles, potentially resulting in prolonged skin exposure and deeper penetration into the dermal layer [77]. Smaller particles can pass through the intercellular lipid matrix or enter straight through epidermal keratinocytes, especially if they are injured [78]. Microplastics that pass through the skin can aggregate on the surface or migrate to lymph nodes; however, there is no compelling evidence that MNPs absorbed dermally enter systemic circulation in large proportions [74], and further research is needed to estimate their quantified absorption. MPs are not only responsible for physical damage (erosion, ulcers, fissures) but are also vectors for infectious pathogens. For example, *Vibrio* spp., *Arcobacter* spp., *Clostridium perfringens*, *Enterobacter* spp., *Escherichia coli*, and *Helicobacter* spp. have been identified on PE MPs in freshwater that come in contact with the dermal barrier (Table 2) [79].

## 6. Influence of MNPs in Propagation of AMR

Recent studies have highlighted the role of MNPs in enhancing the mobility and transfer of ARGs and metal resistance genes (MRGs) in ecosystems, causing the enhanced spread of AMR. Any agent or situation promoting AMR is considered a threat to global public health. A growing body of scientific evidence correlates the elevation of AMR indicators (e.g., HGT, the abundance of ARGs, MGEs) with the presence of persistent MNPs in an ecosystem. However, elaborated information on underlying mechanisms and experimental evidence on the interaction of MNPs with microbes causing the propagation of AMR is still naïve [94,95,96]. The conjugative transfer or HGT of antibiotic resistance genes in *E. coli* is reported to be highly dependent on the size of MPs [97]. UV-aged PS-MPs were found to increase the HGT of ARGs in *E. coli* [98]. MNPs were reported to promote the propagation of ARGs in phosphorus-removing bacteria and induce microbial community shift [99]. MPs also demonstrated selective inhibition of ammonia-oxidizing bacteria and enrichment of nitrite-oxidizing bacteria, leading to partial nitrification [100]. The reports on the interaction of MNPs and microbial communities to date strongly show that MNPs could greatly influence the microbial resistome or changes in the resistomes of environmental niches. Several other reports provide evidence that changes in the resistome profile of different groups of microbes due to the presence of MNPs could further enhance the overall propagation of AMR genes in environmental compartments [101,102].

## 7. MNPs as “Shuttle Trojan Horses” and Their Associated Risks

Due to their surface characteristics, MPs act as “Shuttle Trojan horses” which carry toxic chemicals such as bisphenol A (BPA), different toxic monomers, additives used in plastic manufacturing, infectious agents, parasites, and absorbed/adsorbed contaminants [103]. Toxic chemicals associated with MPs may be heavy metals like arsenic (As), zinc (Zn), copper (Cu), cadmium (Cd), lead (Pb), and chromium (Cr); organic pollutants, polychlorinated biphenyls, polycyclic aromatic hydrocarbons (PAHs), organic pesticides, antibiotics, and oligomers; and also microorganisms such as fungi, diatoms, algae, and pathogenic bacteria that are attached to MNPs [104].

MPs enter the ocean as virgin particles, and then microbial biofilms develop on them with time [105]. Aquatic biofilms (containing fish pathogens, *Aeromonas* spp., *E. coli*, etc.) on MPs serve as reservoirs of antibiotics and stimulate horizontal transfer of clinically important ARGs like *sul1*, *tetA*, *tetC*, *tetX*, *ermE*, *macB*, and *blaTEM* that are present on MPs [106]. Plastic-associated pollutants enter the tissues of different food animals and later become a threat to food safety [107]. Microplastics carry heavy metals and infectious agents (e.g., bacterial, viral, etc.) that cause GIT disturbances, pulmonary neoplasms, obesity, respiratory distress, birth anomalies, cardiovascular disorders, and asthma [108]. For example, Cd promotes cell apoptosis, damage to nucleic acid, skeletal and pulmonary damage, alterations in calcium metabolism, and the formation of renal stones. MPs and associated heavy metals (Cd) affect plant growth and root symbiosis, and also negatively affect soil biodiversity, an indicator of soil fertility [109]. Similarly, Pb can also damage muscles, the brain, and kidneys, and even prove fatal. Polycyclic aromatic hydrocarbons (PAHs) adsorbed by MPs cause cancer, developmental anomalies, and genetic alterations [110].

## 8. MPs Affect Plants, Leading to Shortage of Feed for Animals

### Possible Sources of MPs in Agricultural Soil and Influences of MPs on Soil Structure, Function, Fertility, Soil Microbiota, and Ultimately Plants

**Figure 2:**
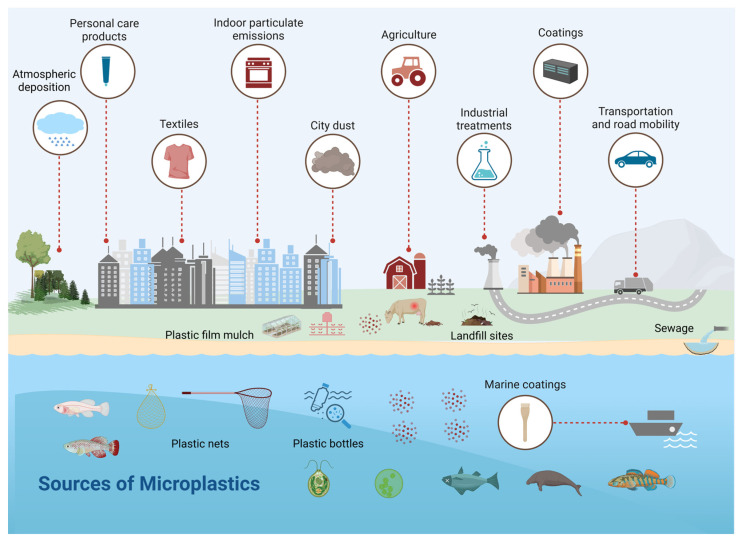
This figure illustrates the flow of MNPs from terrestrial to aquatic ecosystems. Plastics ingested by grazing/indoor animals are hazardous, leading to decreased growth and production and reproductive disorders. Also, through animal food products, these plastic fragments are transferred to consumers through the food chain. Plastics from factories, landfills, human sewage, and agricultural and livestock production systems ultimately enter aquatic ecosystems and then again enter the terrestrial food chain, and this cycle continues.

Organic fertilizer is eco-friendly and a reservoir of soil nutrients [111]. Treated or composted animal dung contains MPs. For example, 150 MPs were detected in 1 kg of pig excrement (14,720 ± 2), 468 plastic particles were detected per kg of chicken manure compost, and 144 particles per kg of goat manure fertilizer were detected [112]. Pesticides used in plastic bottles, plastic processing and recycling plants [112,113], organisms such as earthworms (which play a role in the plastic breakdown) [113], atmospheric deposition of airborne MPs [114], irrigation by wastewater, littering and runoff from the plastic-contaminated surface [113], and the breakdown of the plastic mulch implemented over agricultural lands all add MPs into the soil, alongside other pollutants, e.g., heavy metals [115]. The migration of MPs downward into deeper soil enhances the likelihood of groundwater contamination with MPs and related compounds (Figure 2) [116].

**Figure 3:**
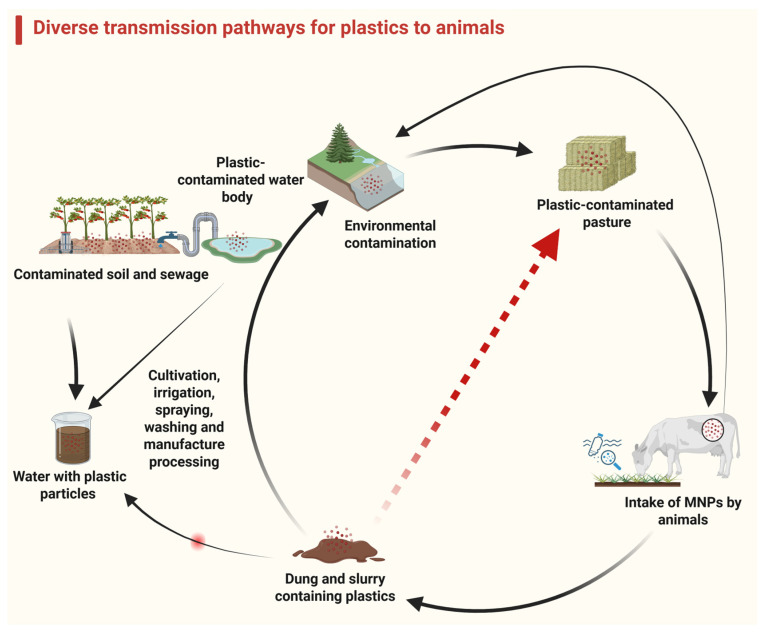
This figure illustrates the various transmission routes of microplastics (indicated by arrows) between animals and the environment, highlighting the role of agricultural practices. Disrupting these transmission pathways can prevent plastic-associated damage in animals.

The roots of plants can absorb MPs, particularly nanoparticles, transferred along the xylem route to edible parts. Polystyrene particles were found scattered throughout the leaf parenchyma [117]. About 80 g out of 400 g of fruit and vegetables that are consumed daily in the developed world contain MPs, which bio-persist and translocate in plants (Figure 3) [32]. In the European Union, 65.5% of people under the age of 15 consume vegetables and fruits every day, consuming high doses of MPs daily. This can be a cause for concern [118].

Microplastics in agricultural soil increase soil porosity [119], which accelerates the evaporation rate, inhibiting the growth of plants [120]. The high-density polyesters effectively decreased the soil’s pH [31]. Microplastics can disturb the diversity and function [121] of the microbial soil community and have a deleterious influence on other soil-based microorganisms [122]. Microplastics inhibit the activity of soil enzymes, for instance, urease, catalase, phenol oxidase, and fluorescein diacetate hydrolase [123]. Higher levels of MPs (28% *w*/*w*) and discharge of dissolved organic compounds such as carbon, nitrogen, and phosphorus [123] lead to a decline in overall agricultural output.

Significant adverse effects of MPs in plants like wheat (*Triticum aestivum*), ryegrass (*Lolium perenne*), fava bean (*Vicia faba*) (reduction of growth, genotoxic and oxidative damage via PS particles of 100 nm) [124], cress (*Lepidium sativum*), and spring onion (*Allium fistulosum*) have been observed [125]. Microplastics and biodegradable MPs (BMPs) potentially prevent plant germination and root and aerial plant development [126].

Depending upon particle size, microplastics produce considerable changes in plant biomass, tissue constituents—such as moisture content, leaf nitrogen level, carbon–nitrogen ratio—root characteristics—like the length of root, mean root dimensions, root tissue stiffness—leaf diameter, and chlorophyll, thereby affecting photosynthetic performance and crop yield [127]. Microplastics as carriers of pathogens and toxic substances negatively impact the development of plant roots and rhizodeposition [128]. Microplastics promote the development of *Hieracium* and *Calamagrostis*, whereas *Holcus* and *Festuca*’s shoot masses have been reduced by microfibers by up to 78% and 51%, respectively (Table 3) [129]. Therefore, further investigation is still needed to determine the negative effects of microplastics on plants, and ultimately on animals.

## 9. Emerging Challenges Associated with MNPs

MNPs are regarded as increasingly relevant particles in air pollution due to their inhalation and interaction with harmful micropollutants, including heavy metals, for instance, Pb, Hg, and Cd [147]. MNPs in the atmosphere may act as important carriers for microorganisms, making their relationship more complex [148]. The interaction of MNPs with pathogens (e.g., bacteria) might exacerbate their harmful effects on living organisms [149]. MNPs have high potential to serve as key carriers of harmful microorganisms, including bacterial pathogens such as antibiotic-resistant bacteria or viruses, which makes them carriers of such diseases [150]. Furthermore, MNPs may accumulate pollutants on their surface, such as hydrophobic organic pollutants, from their surroundings. Based on Lomonaco et al.’s (2020) research work [151], photochemical processes degrade plastic trash, releasing hazardous volatile organic compounds (VOCs) into the atmosphere. This release constitutes a severe hazard linked to the deterioration of plastic waste in the environment.

Plastics’ influence is now recognized as a severe health concern, through two non-exclusive processes. Firstly, MNPs can cause direct neurotoxicity. MNPs can directly affect the central nervous system by percolating through the blood–brain barrier [152], which generates neuronal lesions, decreases synaptic esterases, and fosters proteinopathy and amyloidopathy [153]. Secondly, MNPs can cause indirect neurotoxicity by dysbiogenic impacts, which most likely disturb the microbiota–gut–brain axis (mGBA) [154]. However, the ecotoxicological impact of MNPs has never been investigated in any of these neuropathologies.

MNPs’ impacts on reproduction have also been extensively investigated across many life stages and sexes, with negative consequences such as decreased gametogenesis, reproductive organ damage, altered levels of hormones, and transgenerational impacts [155]. MNPs affect gametogenesis and the development of sperm and eggs. For instance, research investigations on marine invertebrates and small fish models such as zebrafish (*Danio rerio*) demonstrate that MNP exposure can cause structural defects in reproductive cells that result in impaired sperm motility and egg production [155]. Researchers attribute these problems mostly to oxidative damage caused by MPs, which destroy cell membranes and DNA in gametes, potentially leading to reduced fertility [156]. MNPs may affect hormonal balance by interfering with the endocrine system, particularly if they absorb endocrine-disrupting chemicals (EDCs) [157]. Studies have indicated that polystyrene MPs impair hormone production, resulting in altered amounts of estrogen, testosterone, and other reproductive hormones in animals [158]. Low levels of these hormones in mammals can affect spermatogenesis, folliculogenesis, and successful fertilization. Some EDCs have been associated with premature puberty, limited fertility, and unsuccessful pregnancies (Table 4) [159].

## 10. Strategies for Reducing MNPs and Their Potential Hazards

Currently, there are critical initiatives for reducing the prevalence of MNPs and their associated hazards, including technological alternatives and preventative management. Advanced options for eliminating MNPs from the environment often incorporate physical, chemical, and biological technology.

### 10.1. Eco-Sustainability Management Approaches for Reducing Plastic Waste

To reduce ecological damage and health risks, the easiest and most efficient approach is to limit MNP sources. However, the disposal of plastic-based waste is becoming important due to insufficient legislation on long-term disposal methods. To reduce the consequences of MNPs, it is extremely important to effectively handle plastic waste in a cost-effective and environmentally responsible manner [178]. Waste handling can be facilitated by the 4Rs, “Reuse-Reduce-Recover-Recycle”, to reduce plastic pollution [179]. Using upcycling technology, it is feasible to remove MNPs from the waste stream while also creating new goods with greater value and usage [180]. Plastic bottles, for example, can be turned into bird feeders, planters, or structural construction elements. This sort of upcycling enables distinctive and environmentally beneficial artistic creations. Furthermore, Artificial Intelligence (AI)-based systems are now beneficial for handling and decreasing plastic waste and lessening carbon footprint [181].

Numerous works highlight the circular economy as the most effective and viable approach to minimizing plastic pollution and reducing the possible impact of MNPs on living organisms [182]. The circular economy method can prove crucial in boosting an in-depth understanding of optimizing reduction tactics and reducing the harmful environmental impacts of MNPs [183]. Furthermore, life cycle assessment (LCA) concepts are a widely employed approach for identifying and evaluating ecological and biological effects [184]. They are tools that help in environmentally friendly decision-making and proposing alternative methods for protecting habitats, animals, and human well-being. Furthermore, the focus should be on promoting consumer environmental consciousness and education. A continuous approach that requires public understanding and instruction of the MNPs issue and possible responses is needed to accomplish the common aim of reducing plastic waste at its sources or preventing plastic pollution [185].

### 10.2. Technical Ways to Increase the Elimination Efficiency of MNP-Contaminated Waste Streams

MNP elimination techniques include biodegradation, composting, recycling, and thermal processing. Biodegradation uses enzymes and microorganisms to break down and mineralize MNPs [183]. After mineralization, the plastic derivatives are transported into the cytoplasm of microbes and further metabolized, yielding chemicals such as N2, CO_2_, and H_2_O. Additionally, numerous enzymes have been widely employed to degrade synthetic MP polymers [186]. Overall, biodegradation techniques are usually an effective way to reduce MNP levels while needing minimal energy input and showing potential performance [183]. Many studies have proven the degradability of MNPs by biological species such as fungi and bacteria [187], algae, earthworms [188], and snails [189]. The findings also demonstrate that hyper-thermophilic bacteria strains play an important role in MP biodegradation during hyper-thermophilic composting (hTC), indicating that hTC has the potential to be used to remove MPs [190]. This demonstrates that microorganisms, including fungi, bacteria, mealworms, algae, etc., can be used to biodegrade MPs and successfully reduce plastic contamination.

## 11. Suggestions for Setting an Animal Health Hazard Assessment Index for MNP Exposure

Developing a robust animal health hazard assessment index for MNP exposure necessitates an interdisciplinary approach, integrating exposure science, toxicokinetics, toxicodynamics, and environmental detection. Based on the current literature, the following suggestions are integral:

- Multi-Route Exposure Quantification

Exposure evaluation should distinctly consider the three key routes (inhalation, ingestion, and dermal contact), as each has distinct absorption and toxicological profiles (Table 1). Hence, exposure doses can be quantified by combining environmental monitoring data (quantities of MNPs in water, feed, soil, and air) with species-specific behavioral and ecological information [52].

- Species-Targeted Toxicokinetics and Toxicodynamics

The index should include species-specific variations in absorption, distribution, metabolism, and excretion (ADME) of MNPs. Physiological characteristics such as gut morphology, respiratory architecture, and dermal barrier integrity significantly affect MNP uptake and systemic effects (Table 1) [52].

- Effect-Based Biomarkers

These include bioindicators that reflect oxidative damage, inflammatory cascades, immune dysfunction, reproductive issues, and metabolic disruptions. Molecular markers, such as gene expression, that vary in stress response and detoxification pathways, can enhance sensitivity and specificity in hazard detection and quantification [49,191].

- Bioaccumulation and Trophic Transfer

This includes evaluating the accumulation of MNPs and linked chemicals in animal tissues, as well as potential biomagnification through food webs, which can worsen hazard severity [49,52,191].

- Chemical Additives and Adsorbed Pollutants

Researchers should integrate the potential hazards associated with plastic additives (e.g., bisphenols, phthalates) and adsorbed foreign contaminants (pathogens, heavy metals) carried by MNPs, as these can exaggerate toxicity [49].

- Composite Hazard Scoring System

Researchers should develop a weighted scoring system that integrates exposure dose, toxicological endpoints, and species sensitivity to generate an overall hazard index. This system should be adaptable to different environmental contexts and animal species [192].

- Probabilistic Risk Assessment

The index should apply probabilistic models to incorporate variability and uncertainty in exposure and response, enhancing the reliability and robustness of hazard assessments [193,194].

- Chronic and Cumulative Effects

Researchers should include long-term, low-dose exposures and cumulative impacts with other environmental stressors to better reflect real-world scenarios and chronic health risks [194].

## 12. Conclusions and Future Research Considerations

This review identifies MNPs as an important consideration and discusses their detrimental impact on plants, and aquatic and terrestrial animals. It also explains the mechanisms of potential MNP influences via ingestion, inhalation, and dermal routes, as well as future challenges and opportunities for MNP waste management. The study of MNP pollution has attracted global interest, and some progress has been made in certain areas. While there are still many difficulties with this growing pollution, this review’s findings provide current and thorough information regarding the properties of MNPs, which may be used to promote future research trends. Based on these findings, various strategies, such as filters, barriers, and clean-up efforts, have been developed to mitigate macro- and microplastic pollution. However, no established methods currently exist for addressing nanoplastic pollution. This represents a critical gap in the literature, emphasizing the need for further research and innovative solutions.

At present, MPs pose an imminent danger to the environment. Studies on exposure-related toxicity in conjunction with harmful chemicals are critical for comprehending MP characteristics and establishing a mitigation plan. Sensitivity and toxicological impacts on aquatic and freshwater creatures are characterized by exposure dosage as either extremely high (resulting in lower reproductive output, organ damage, and mortality) or low (resulting in behavioral changes over time). According to reports, toxicity research for people is still ongoing, but plants and animals have received less attention. It is suggested that if a study conducted an evaluation of co-exposure to MNPs and chemical pollutants, the experimental setup must enable some form of contrast between individual and combination hazards [185], because comparing these studies is still challenging.

It is critical to design easy, precise, effective, and inexpensive techniques. A more extensive study is required to control MNP pollution and assist local governments with making decisions and associated policies. For example, screening roadways and residential dust should be regarded as a low-cost strategy for MNP contamination in residential regions. As previously noted, additional efforts must be taken to establish international standards, attempt to record health impact assessments, and develop policy frameworks that may be assessed in the surrounding air.

Finally, regulations for plastic prevention, waste stream control, and alternative eco-friendly resources should be promoted. To effectively tackle the environmental implications of MNPs, it is critical to set legislation and standards governing plastic use and dumping. Studies can give scientific data that will encourage the formulation of these rules and ensure that they are centered on environmental sustainability and health concerns. MNPs are capable of being biodegraded and transformed into CO_2_ and H_2_O, hence protecting ecosystems. It is important to establish an important cycle that lowers MNPs release at sources. In terms of the circular economy, the establishment of integrated techniques to transform MPs into microbial polymers, which may be used and processed by microorganisms into innovative biopolymers, is worth investigating [183,195,196,197,198]. However, it should be noted that such technologies are still in the early developmental stages and far from being commercialized. Recent studies highlight the technical and economic hurdles that crucially must be overcome before these processes can be implemented at a commercial scae [187].

Recent studies suggest that MNPs can lead to alterations in the gut microbiota of animals [57,58], potentially affecting enteric absorption and immune response, an area that remains largely unexplored currently. In addition, the role of MNPs in facilitating the transfer of antimicrobial resistance genes within livestock is an emerging concern with significant implications in both animals and the public health sector, so it can be focused on in future research.

## Figures and Tables

**Table 1:** Differences in hazards between animals and humans concerning exposure routes.

Exposure Route	Humans: Main Hazards	Animals: Main Hazards	Key References
Ingestion	- Gut barrier breach and systemic translocation to organs - Immune dysfunction and enteritis - Gut microbiome dysbiosis - Metabolic disorders like impaired lipid and glucose metabolism - Potential genotoxicity and carcinogenicity - Accumulation of plastic-linked chemicals like bisphenols and phthalates	- Accumulation in gills, kidney, gut, liver (aquatic) - Oxidative injury and inflammation - Energy metabolism disruption - Reproductive toxicity (reduced fertility, abnormal development) - Alteration in feeding behavior and nutrient absorption - Vector for antimicrobial resistance genes and pathogens	[54,55,56,57,58,59,60,61,62,63,64,65,66]
Inhalation	- Respiratory issues like fibrosis, inflammation - Translocation to systemic circulation - Neurotoxicity - Allergic and asthmatic issues - Exacerbation of pre-existing lung pathology	- Respiratory tract inflammation and lesions - Systemic distribution via bloodstream - Behavioral changes (in rodents) - Reduced growth and survival (in aquatic larvae) - Potential for bioaccumulation in food chains	[61,67,68,69,70,71]
Dermal	- Generally low systemic uptake - Local irritation, inflammation, or allergic reactions (especially with damaged skin) - Penetration enhanced for nanoparticles (<100 nm), especially via hair follicles or wounds - Potential for carrying toxic additives or pathogens	- Local skin lesions and irritation - Increased risk with frequent soil/water contact or damaged/thin skin (hide) - Potential vector chemicals and pathogens - No strong evidence for systemic translocation, but risk is higher in species with thinner skin or frequent exposure	[70,72,73,74,75,76,77,78,79]

**Table 2:** Effects of MNPs exposure on aquatic organisms and associated details.

Organism	Plastic Type and Size	MPs Concentration	Suspension	Detection Method and Sample Analyzed	Effects on Aquatic Animals	Reference
Zebrafish (*Danio rerio*) (*n* = 20)	PS * and BSA *, 100 nm, 5 μm, and 200 μm	10 g/L	Freshwater	Microscope, intestine	Altered gene expression and signaling processes of macrophages, impaired food consumption, and impaired function of Keap1-Nrf2-ARE signaling pathway in intestine	[80]
Oyster (*Ostreidae*) (*n* = 25)	PS, 50–500 nm and 2 μm	0.1, 1, 10, and 25 µg/mL	Ultrapure water	Raman microspectroscopy, gametes, embryos, and larvae	Reduced fertilization and larval hatching, genotoxicity	[27]
Mussels (*Mytiulus galloprovincialis*) (*n* = 30)	PS, 110 nm	0.005, 0.05, 0.5, 5, and 50 mg L^−1^	Artificial saltwater	NanoDrop spectrophotometer, digestive glands, gills, and hemolymph	Affects glutathione transferase and isocitrate dehydrogenase activity, increased atresia of oocytes	[81]
Mollusk (*Tegillarca granosa*) (*n* = 720)	PS, 500 nm and 30 μm	25 mg/mL	10 mL aqueous suspension without additives	HPLC *, immune system, and body tissues	Decrease total hemocyte count, phagocytosis, and ATP content, and increase Caspase 3 activity; increase immunotoxicity, disturbing neuroendocrine system as well	[82]
European sea bass (*Dicentrarchus labrax*) (*n* = 6)	PVC *, PE *, 40–150 μm	1, 10, and 100 mg mL^−1^	Seawater aquaria (250 L)	qPCR *, respiratory system	Decrease phagocytic capacity; increase respiratory burst activity of HKLs, affecting the immune cells.	[83]
Nematodes (*Caenorhabditis elegans*) (*n* = 30)	PS, 1.0–5.0 μm	1 mg L^−1^	K-medium *	Stereomicroscope and Motic microscope, intestine, and physical parameters	Reduced body length, survival rate, overall life span; raised number of head thrashes, mRNA gene expressions, and crawling speed	[84]
Fish (*Danio rerio*) (*n* = 100)	PS, 5 μm	20 and 200 μg/L	Ultrapure water	qRT-PCR, zebrafish tissues (livers, guts, and gills)	Increased accumulation in gill, intestines, and liver; infiltration in hepatocytes; cilia abnormalities in enterocytes; and fuzzy structure in gill filament cells	[85]
Juvenile intertidal fish (*Girella laevifrons*) (*n* = 30)	PS, 8 μm	0.15 g	Pellets	Microscope, intestine	Histopathological alterations, including leukocyte infiltration, villus cell death, and hyperemia leukocyte infiltration	[86]
Shellfish (*Argopecten irradians*) (*n* = 40)	PS, 1 μm	10, 100, and 1000 beads/mL	80 L of aerated and filtered seawater	Fluorescence microscope, digestive tract	Increased functioning of antioxidant enzymes H_2_O_2_, SOD, and CAT, inducing oxidative damage in bivalves; increasing oxidative stress may result in histological deterioration	[87]
Zebrafish (*Danio rerio*) (*n* = 270)	MPs, 20–200 μm	20 mg/L	Culture water	Fluorescence spectrometer, microscope, intestine	Decreased mucus secretion and D-lactate levels, increased superoxide dismutase activity, caused mucosal injury, enhanced permeability, inflammation, metabolic disturbance, and microbial dysbiosis	[88]
Medaka (*Oryzias. melastigma*) (*n* = 5)	PS, 50 nm and 45 μm	10 μg/mL	Artificial sea water	DNA extraction Gut and liver tissue	Increased mucus secretion, gut D-lactate levels, and gut diamine oxidase levels; increased ROS and decreased SOD and CAT	[89]
Juvenile guppy (*Poecilia reticulata*) (*n* = 180)	PS, 32–40 μm	100 μg/L (MP-100) and 1000 μg/L	Freshwater	Spectrophotometer, PCR, intestine	Decreased digestive activity of enzymes, elevated goblet cell production and gut secretion of TNFα, IL6, and IFNγ; impaired digestive performance and induced microbiota dysbiosis in gut	[90]
Mussel (*Mytiulus galloprovincialis*) (*n* = 32)	PS, 110 nm	0.005, 0.05, 0.5, 5, and 50 mg L^−1^	Artificial saltwater	Microscope, digestive glands, and gills	Elevated Hsp70 mRNA levels in digestive glands, lipid peroxidation, total oxidant status, RNA damage; influenced cell-tissue repair and immune system	[81]
Juvenile isopods (*I. emarginata*) (*n* = 22)	PS, 10 µm	10 mL	Demineralized water (aqua dem.)	Microscope, gut	Do not block gastrointestinal organs of isopods and do not have harmful impacts on their life history aspects	[91]
Brine shrimp (*Artemia franciscana*)	PS and NPs *, 40 nm	50 and 100 mg/ml	Natural sea water	Fluorescent microscope, gut	Impaired food uptake, motility, and multiple molting of brine shrimp larvae	[92]
Beachhoppers (*Platorchestia smithi*)	PE, 38–45 µm	1 μL	NSW	Gas chromatograph	Impact on survival and behavior, reduced nutrition, and decline in capability of individuals to respond to diverse biotic and abiotic signals	[93]

(* = The full form/explanation of this abbreviation is given in the footnote of the table). PS (polystyrene), PE (polyethylene), PVC (polyvinyl chloride), NPs (Nanoplastics), MPs (microplastics), HPLC (high-performance liquid chromatography), qRT-PCR (quantitative real-time PCR), K-medium (32 mmol L^−1^ KCl, 51 mmol L^−1^ NaCl).

**Table 3:** Toxic effects of MNPs on terrestrial plants and associated details.

Plants Specie	Plastic Type and Size	Concentration	Exposure	Culture Method	Effects on Plants	Reference
Corn (*Zea mays* L.)	PE *, 212–300 μm	0.1% (*w*/*w*)	28 days	Soil culture	Adversely impact maize and ecology of soil bacteria, and influence antioxidant gene expression	[130]
Lettuce (*Lactuca sativa*)	PMFs *, 2.55 mm	0.1%, 0.2% (*w*/*w*)	59 days	Soil culture	Negatively impacted plant shoot length, photosynthesis, and chlorophyll content, and altered nitrogen and carbohydrate content as well.	[131]
Rice (*Oryza sativa* L.)	PS *, 135.9–530 μm	0.01%, 0.5% (*w*/*w*)	142 days	Soil culture	Affected metabolite accumulation and energy expenditure of rice	[132]
Cabbage (*Brassica oleracea*)*,* Radish (*Raphanus sativus cv.*)	PE *, <1 mm	0.01, 0.1, 1, 10, 100, 1000, 10,000 mg/L	12 days	Hydroponics	Induce oxidative stress in radish buds, which leads to increase in ROS *, inhibits nutrient absorption by seedlings, and increases anthocyanin content	[133]
Rapeseed (*Brassica napus* L.)	PE, 293 μm	0.001%, 0.01%, 0.1% (*w*/*w*)	60 days	Soil culture	Reduced overall chlorophyll concentration in cabbage and influenced sugar synthesis, increased accumulation of heavy metals, and reduced beneficial components in rapeseed plants	[134]
Pumpkin (*Cucurbita pepo* L.)	PP, PE and PVC *, 40–50 μm	0.02%, 0.1%, 0.2% (*w*/*w*)	28 days	Soil culture	Impaired root and especially shoot growth; reduction in leaf size, chlorophyll content, and photosynthetic efficiency	[127]
White clover (*Trifolium repens*), and rose balsam (*Impatiens balsamina*)	PS, 2 μm, 80 nm	0, 10, 50, 100, 500 mg/L	7 days	Hydroponics	Germination rates of these plants dropped significantly as PS concentration increased	[135]
Lettuce (*Lactuca sativa* L.)	PS, 0.1–1 μm, >10 μm	0.25, 0.5, 1 mg/mL	28 days	Hydroponics	Caused physical blocking of root pores, produced phototoxicity, and drastically reduced MBP * and DBP * accumulation in leaves, lettuce, and roots.	[136]
Barley (*Hordeum vulgare* L.)	PS (5.64 ± 0.07 μm), PMMA * (96.75 ± 0.58 nm)	2 g/mL	14 days	Hydroponics	Limited development of rootlets, elevated ROS levels, and altered functions of ROS metabolism enzymes in roots and leaves	[137]
Wild carrot (*Daucus carota*)	PA, PP, LDPE and PS, <5 mm or <5 mm^2^	0.1%, 0.2%, 0.3%, 0.4% (*w*/*w*)	28 days	Soil culture	All shapes of MPs boosted plant biomass	[138]
Cucumber (*Cucumis sativus* L.)	PS, 100, 300, 500, 700 nm	50 mg/L	21 days	Hydroponics	Transfer of PS to underground section through cucumber stem, where PS decomposition may release benzene, hence impacting chlorophyll and carbohydrate metabolism	[139]
Greater Bladderwort (*Utricularia vulgaris*)	PS, 1, 2, 5 μm	15, 70, 140 mg/L	7 days	Hydroponics	Caused changes in composition of pigment and protein, and produced severe ecological toxicity and oxidative damage	[140]
Onion (*Allium cepa* L.)	PS, 100 nm	25, 50, 100, 200, 400 mg/L	30 days	Hydroponics	Significantly decreased root length and produced cytogenetic toxicity by enhancing production of ROS and suppressing cdc2 expression	[141]
Maize (*Zea mays* L.)	PE, 3 μm	0.0125, 100 mg/L	10, 15 days	Hydroponics	Caused significant decrease in nitrogen content, transpiration, and production of maize	[142]
Red rice (*Oryza sativa* L.)	PS, PTFE *, 10 μm	0.04, 0.1, 0.2 g/L	10 days	Hydroponics	High concentration lowered rice biomass, gross photosynthetic rate, and chlorophyll concentration, and produced oxidative burst in rice tissues	[143]
Wheat (*Triticum aestivum* L.)	PS, 100 nm and 5 μm	Hydroponics: (0, 10, 20, 50, 100, 200 mg/L), soil culture: (0, 1, 10, 50, 100 mg/kg)	Soil: 6 days, culture: 10 days	Soil culture and hydroponics	In hydroponics test, excessive concentrations reduced wheat root and stem elongation; in condition of soil culture, photosynthesis of wheat leaves impaired, and biosynthesis of protein hampered	[144]
Perennial ryegrass (*Lolium perenne*)	HDPE *, PLA *, 102.6 μm	0.001%, 0.1% (*w*/*w*)	30 days	Soil culture	Decreased germination rate of seedlings and height of shoots	[145]
Duckweed (*Lemna minor*)	PE, 4–12 μm	0, 10, 50, 100 mg/L	7 days	Hydroponics	Inhibited root development and impaired viability of root tissue cells	[146]

(* = The full form/explanation of this abbreviation is given in the footnote of the table). PS (polystyrene), PE (polyethylene), PP (polypropylene), PVC (polyvinyl chloride), MPs (microplastics), ROS (reactive oxygen species), HDPE (high-density PE), LDPE (low-density PE), PMMA (polymethyl methacrylate), PMFs (polyester microfibers), PLA (polylactic acid), PTFE (polytetrafluoroethylene), DBP (di-butyl phthalate).

**Table 4:** Adverse effects of MNPs on different terrestrial animals and associated case details.

Mammals	Plastic Type and Size	Concentration	Exposure Days	Route	Adverse Effects	Reference
Male ICR mice	PS *, 0.5 and 50 mm	0.024 and 0.24 mg/kg/day	5 weeks	Replacement of water in drinkers with PS suspension	Decrease in BW *, decreased Klf4 and Muc1 expression in colon and mucin secretion, considerable modification in composition of intestinal microbiota, and lipid metabolism abnormalities in liver	[160]
Sheep	MPs *, 10 to 100 μm	2 × 10^3^ particles kg^−1^	N/A	Via soil	Caused digestive disorders, including persistent ruminal tympany and gastrointestinal blockage, resulting in impaired development	[161]
ICR mice	PE *, 40–48 mm	3.75, 15, and 60 mg/kg/day	13 weeks	Infusion of PE * suspension through a gastric tube	MP-induced immune system reactions in animals: In both sexes of mice, proportion of neutrophils and IgA in blood was boosted in females, and subpopulation composition of lymphocytes in spleen changed; in animals administered with MP, percentage of live births per female reduced dramatically	[162]
Lambs	MPs, 25 μm, 50 μm and 100 μm	100 mg/day	56–60 days	Supplemented with diet	Inhibited lambs’ digestive function, significantly impacted blood and organ health status, decreasing daily average growth, decreasing hemoglobin and levels of albumin in lamb blood, initiating oxidative stress, and causing serious harm to jejunum	[163]
Male C57 mice	PS, 5 mm	0.12 mg/kg/day	7 days	Replaced water in drinkers with MP concentration	Caused acute colitis, produced dystrophic alterations in liver	[164]
Goat	MPs, 5 mm	0∼1.6 mg/mL	N/A	In diet	Decreased modified cell morphology and cell viability, disrupted organelle integrity, caused mitochondrial dysfunction and oxidative stress	[165]
Pig	BPA, 3 mm	3 nM, 300 nM, or 30 μM	48 h	In vitro culture	Effected oocyte maturation, cytoskeletal disruption, changed mRNA levels and cumulus expansion.	[166]
Male Balb/c mice	PS 5–5.9 mm	0.4; 4 and 40 mg/kg/da	6 weeks	Administration of MP suspension through a gastric tube	MPs reduce quantity and motility of spermatozoa, increase percentage of malformed spermatozoa, decrease activity of succinate dehydrogenase and lactate dehydrogenase, lower testosterone levels, and lead to oxidative stress	[167]
Male and female Sprague Dawley rats	PS, 0.1 mm (NP)	0.75 × 10^5^, 1.5 × 10^5^ and 3 × 10^5^ particles/sm^3^	12 weeks	Inhalation	Increase in overall weight of heart, drop in quantity of lymphocytes and leukocytes in blood, and decline in inspiration time; additionally, TGF-β *, TNF-α *, and cytokine levels shown to rise in lung tissue	[168]
Male C57/B6 mice	0.07 mm (NP) and 5 mm	0.2 and 2 mg/kg/day	4 weeks	Suspension through gastric tube	Caused damage to gastrointestinal tract, reduction in expression of tight junction proteins in intestine epithelium, and significant alterations in intestinal microbiota	[169]
Female Wistar rats	PS, 0.5 mm	0.06, 0.6, and 6 mg/kg/day	13 weeks	Replaced water in drinkers with MP concentration	MPs identified in ovarian granulosa cells induce apoptosis and occurrence of ovarian fibrosis	[170]
Cattle	BPA, 1400 um	5.8–105.8 ppb and 3.3–30 ppm	Water bodies, soil, and atmosphere	Food, water, inhalation (air), and dermal contact	Reduced cleavage rate and embryo development at blastocyst stage along with modifications to gene expression in cattle	[171]
Male ICR mice	PE, 5 mm	0.024, 0.24, and 2.4 mg/kg/da	5 weeks	Replaced water in drinkers with MP concentration	Less viable spermatozoa in epididymis and higher levels of pro-inflammatory markers (IL-1β * and NF-κB *)	[172]
Male C57BL/6 mice (30)	PS, 1–10 mm and 50–100 mm	2.4 mg/kg/day	8 weeks	Replaced water in drinkers with MP concentration	Alterations in intestinal mucosa affect intestinal flora of hares with inflammation and development of acute intestinal infection, as well as modifications in intestinal mucosa, leading to loss of weight, diarrhea, and death	[173]
Male Swiss mice	PS 0.023 mm (NP)	14.6 ng/kg	3 days	Intraperitoneal injection of MP	MPs cause cognitive deficits, violations of redox balance, and reduction in acetylcholinesterase activity in brain	[174]
Male CD-1 mice	PE, 0.4–5 mm	100 mg/kg/day	4 weeks	Suspension through gastric tube	MPs can penetrate mice’s testes, and MPs containing phthalates accumulate in the testes, liver, and intestines, also increasing reproductive toxicity	[175]
Pregnant female rat	PS, 0.02 mm	2.64 × 10^14^ MP particle	19th day	Intratracheal administration	Influences heart, maternal lungs, and spleen; found in lungs, liver, placenta, heart, brain, and kidneys of fetuses, indicating MP translocation from mother’s lungs to fetal tissue in late pregnancy	[176]
Male C57BL/6 mice	PE 10–150 mm	0.24, 2.4, and 24 mg/kg/day	5 weeks	MP in food	Alteration in composition of intestinal microbiota, rise in amount of IL-1a * in blood serum, and increase in amount of Treg cells and Th17 * all produce inflammation in intestinal tract	[177]

(* = The full form/explanation of this abbreviation is given in the footnote of the table). PS (polystyrene), PE (polyethylene), PET (polyethylene terephthalate), PP (polypropylene), PVC (polyvinyl chloride), MPs (microplastics), BW (body weight), TGF-β (Transforming growth factor-β), IL-1 (interleukin-1 alpha), Th17 (T helper type 17), NF-κB (Nuclear factor kappa B), IL-1β (interleukin-1-beta), TNF-α (tumor necrosis factor-alpha).

## Data Availability

No new data were created or analyzed in this study because this study was a comprehensive review.
